# Two new genera with species of the tribe Sarimini (Hemiptera, Fulgoromorpha, Issidae) from China

**DOI:** 10.3897/zookeys.956.47784

**Published:** 2020-08-06

**Authors:** Zhi-Min Chang, Lin Yang, Xiang-Sheng Chen

**Affiliations:** 1 Key Laboratory of Animal Genetics, Breeding and Reproduction in the Plateau Mountainous Region, Ministry of Education, College of Animal Science, Guizhou University, Guiyang, Guizhou, 550025, China Guizhou University Guiyang China; 2 Institute of Entomology / Special Key Laboratory for Developing and Utilizing of Insect Resources, Guizhou University, Guiyang, Guizhou, 550025, China Guizhou University Guiyang China; 3 The Provincial Key Laboratory for Agricultural Pest Management of Mountainous Regions, Guizhou University, Guiyang, Guizhou, 550025, China Guizhou University Guiyang China

**Keywords:** Female genitalia, issid, morphology, Oriental region, planthopper, taxonomy

## Abstract

*Tempsarima* Chang & Chen, **gen. nov.** (Hemiptera: Issidae: Sarimini), with type species *Tempsarimabipunctata* Chang & Chen, **sp. nov.** and *Tetrichina* Chang & Chen, **gen. nov.** (Hemiptera: Issidae: Sarimini), with type species *Tetrichinatrihamulata* Chang & Chen, **sp. nov.** are described and illustrated from Hainan Province of China. The female genitalia characters of Issidae are discussed.

## Introduction

The new classification of Issidae Spinola, 1839 with three subfamilies (Wang et al. 2016), recently updated to four subfamilies (Zhao et al. 2019), currently groups nine tribes (Gnezdilov 2018, 2019a; Bourgoin 2020) in which hindwing characters appear to be important for issid systematics. In this frame, several new taxa were established (Wang et al. 2017; Gnezdilov 2018, 2019a; Chang et al. 2019; Zhao et al. 2019). However, due to the classification by Wang et al. (2016) based on limited taxa analysis, many unverified taxa had to be placed in *incertae sedis* position in the classification (Bourgoin 2020) and needed to be re-examined.

**Figures 1–4.**
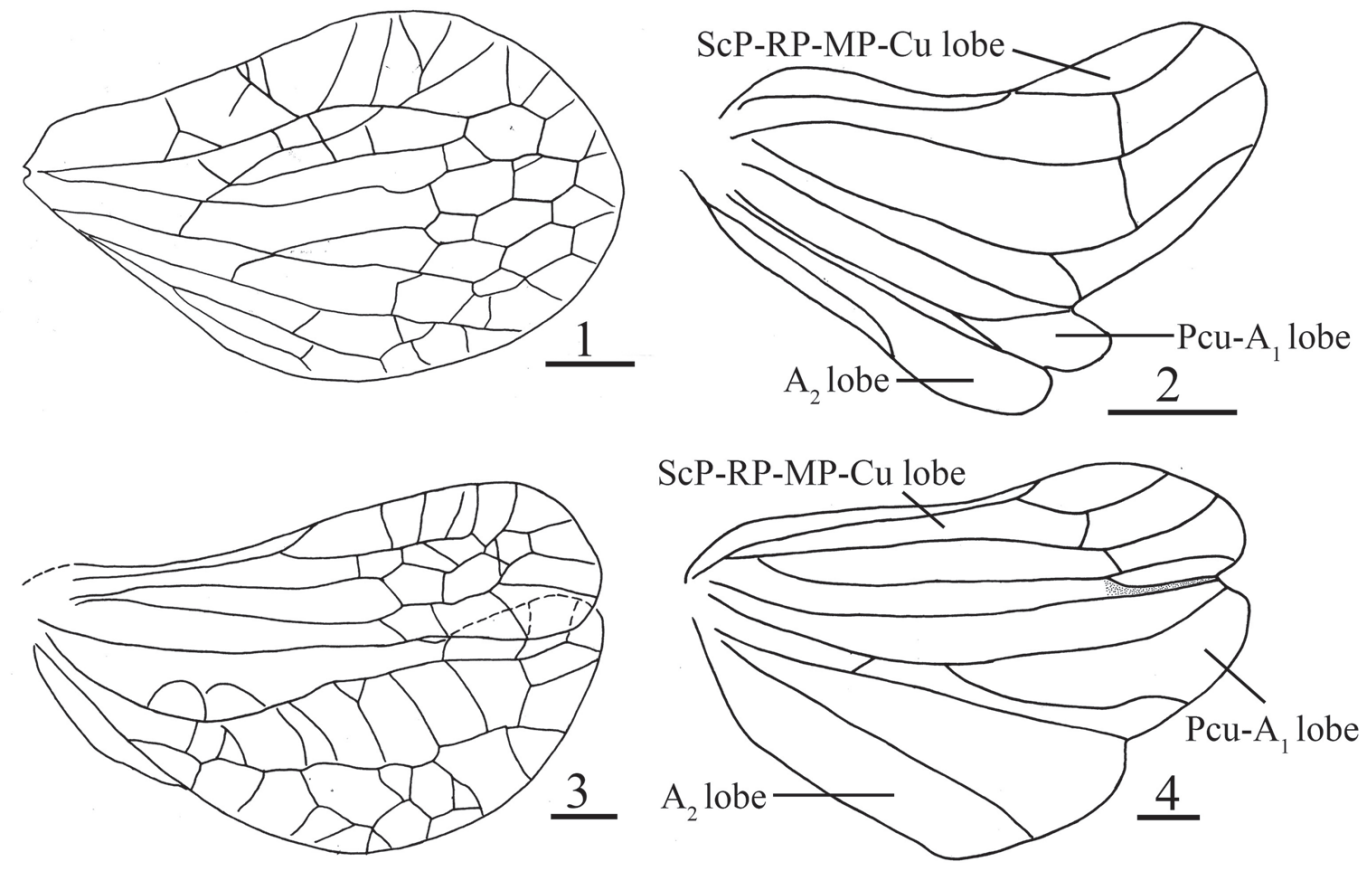
Hindwing **1***Ishiharanusiguchii* (Matsumura, 1916) **2***Kodaianellafurcata* Chang & Chen, 2020 **3***Tetricodessimilis* Chang & Chen, 2017 **4***Longieusarimalunulia* Wang, Bourgoin & Zhang, 2017. Scale bars: 0.5 mm.

The subfamily Hemisphaeriinae Melichar, 1906 sec. Wang et al. (2016) contains Hemisphaeriini Melichar, 1906, Kodaianellini Wang, Zhang & Bourgoin, 2016, Parahiraciini Cheng & Yang, 1991, and Sarimini Wang, Zhang & Bourgoin, 2016, all of Oriental, Australian, and Afrotropical region origin. The characters of hindwings as distinguishing features, make it easier to quickly recognize and define these groups (Wang et al. 2016): Hemisphaeriini, a single lobe, short and reduced or absent (Fig. 1); Kodaianellini, three lobes, with Pcu-A_1_ lobe distinctly thinner, less than half as wide as ScP-RP-MP-Cu lobe, A_2_ lobe with anterior and posterior margins subparallel (Fig. 2); Parahiraciini, two lobes, with veins network-like, a deep narrow incision in apical margin of hindwing, Pcu-A_1_ lobe distinctly wider than ScP-RP-MP-Cu lobe, A_2_ lobe short, thin or absent, Pcu and A_1_ free, not partially fused (Fig. 3); and Sarimini, three developed lobes, Pcu-A_1_ lobe more or less as wide as ScP-RP-MP-Cu lobe and Pcu single or branched, Pcu and A_1_ anastomosing for a shorter or longer distance, A_2_ not branched (Fig. 4). The tribe Sarimini now contains 25 genera (Bourgoin 2020), including two recently described new genera *Microsarimodes* Chang & Chen, 2019 (Chang et al. 2019), and *Eusarimissus* Wang & Bourgoin, 2020 (Wang and Bourgoin 2020), and the transfer to the tribe Sarimini of eight genera previously in *incertae sedis* position: *Balisticha* Jacobi, 1941, *Euroxenus* Gnezdilov, 2009, *Givaka* Distant, 1906, *Neosarima* Yang, 1994, *Sinesarima* Yang, 1994, *Sundorrhinus* Gnezdilov, 2010, *Tempsa* Stål, 1866 and *Yangissus* Chen, Zhang & Chang, 2014 (Bourgoin 2020; Chang et al. 2019; Constant and Bartlett 2019; Wang et al. 2019). Currently, fourteen genera of Sarimini are recorded in China: *Euroxenus* Gnezdilov, 2009, *Eusarima* Yang, 1994, *Eusarimissus* Wang & Bourgoin, 2020, *Longieusarima* Wang, Bourgoin & Zhang, 2017, *Microsarimodes* Chang & Chen, 2019, *Neosarima* Yang, 1994, *Nikomiklukha* Gnezdilov, 2010, *Orbita* Meng & Wang, 2016, *Parasarima* Yang, 1994, *Sarima* Melichar, 1903, *Sarimodes* Matsumura, 1916, *Sinesarima* Yang, 1994; *Yangissus* Chen, Zhang & Chang, 2014, and *Tetrica* Stål, 1866 (Chan and Yang 1994; Chang et al. 2019; Chen et al. 2014; Gnezdilov 2019b; Meng et al. 2016; Wang and Bourgoin 2020; Wang et al. 2017, 2019).

While sorting out the specimens from Hainan Province (China), we found two new genera and species belonging to the tribe Sarimini, which are described here: *Tempsarima* Chang & Chen, gen. nov. with type species *T.bipunctata* Chang & Chen, sp. nov. and *Tetrichina* Chang & Chen, gen. nov. with type species *T.trihamulata* Chang & Chen, sp. nov. In addition, the female genitalia of *T.bipunctata* Chang & Chen, sp. nov., as a special type of female genitalia in Sarimini, is recorded and discussed.

## Materials and methods

The morphological terminologies follow Chan & Yang (1994) for the head and body, Bourgoin et al. (2015) for the wing venation, and Bourgoin (1987, 1993) and Gnezdilov (2002, 2003) for male and female genitalia respectively. Dry specimens were used for descriptions and illustrations. The genital segments of the examined specimens were macerated in 10% NaOH, washed in water and transferred to glycerine. Illustrations of the specimens were made with a Leica M125 and Olympus CX41 stereomicroscope. Photographs were taken with KEYENCE VHX-1000C and KEYENCE VHX-6000C.

The examined specimens are all deposited in the Institute of Entomology, Guizhou University, Guiyang, China (**IEGU**).

## Taxonomy

### Family Issidae Spinola, 1839

**Subfamily Hemisphaeriinae Melichar, 1906 sec.**

**Tribe Sarimini Wang, Zhang & Bourgoin, 2016**

#### Tempsarima

Taxon classificationAnimaliaHemipteraIssidae

Chang & Chen gen. nov.

1AE5A8AE-636F-552E-A71D-5C2BD932D8FF

http://zoobank.org/565A78E1-D30B-4CDE-8414-D2A6157A8D9F

Figures 5–16 , 17–27

##### Type species.

*Tempsarimabipunctata* Chang & Chen, sp. nov., here designated.

##### Diagnosis.

**Figures 5–16.**
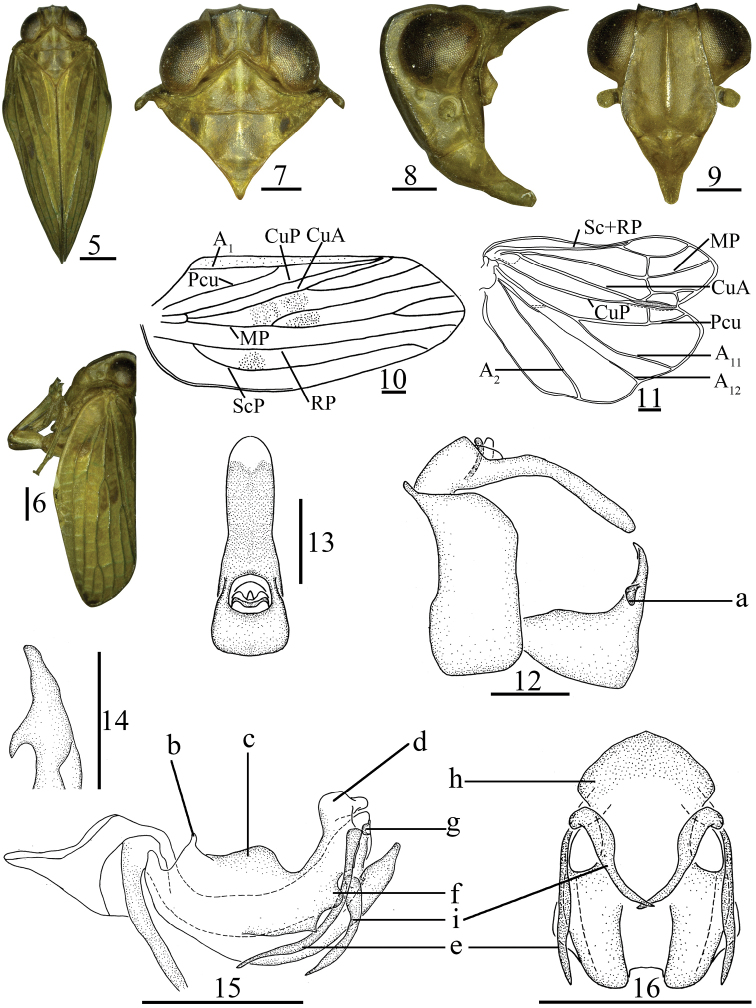
*Tempsarimabipunctata* Chang & Chen, sp. nov. **5** adult, dorsal view **6** same, lateral view **7** head and thorax, dorsal view **8** same, lateral view **9** head, ventral view **10** forewing **11** hindwing **12** male genitalia, lateral view **13** male anal segment, dorsal view **14** capitulum of genital styles, posterior view **15** aedeagus and phallobase, lateral view **16** same, ventral view. Scale bars: 0.5 mm. Abbreviations: a–irregular triangular prominence, b–small claviform process, c–convex protrusion, d–duck mouth-liked process, e–long hooked process, f–lobe-like process, g–small lamina-like process, h–mushroom-liked, i–short hooked process.

This genus is similar to the genus *Sarimodes* Matsumura, 1916, but it differs from the latter by: 1) frons smooth (Fig. 9) (frons with verrucae along lateral margin and basal part in *Sarimodes* (Meng and Wang 2016: fig. 18)); 2) forewing with ScP vein long, reaching apical margin, and MP vein forked before the middle of forewings (Fig. 10) (forewing with ScP vein surpassing the middle of forewing, but not reaching apical margin; MP vein forked near distal part in *Sarimodes* (op. cit.: fig. 19)); 3) male genitalia with genital styles irregularly triangular in lateral view; anterodorsal and ventral margins parallel (Fig. 12) (genital styles irregularly rounded, dorsal and ventral margins not parallel in *Sarimodes* (op. cit.: fig. 22)); 4) apical part of dorsal lobe of phallobase with hooked process in lateral view (Fig. 15) (with sword-like process in *Sarimodes* (op. cit.: fig. 24)); 5) female anal tube and genitalia strongly developed and elongate, saw-like (Fig. 18) (not as above in *Sarimodes* (op. cit.: figs 28, 31)).

##### Description.

Body medium in size.

***Head and thorax*.** Width of head including eyes obviously narrower than pronotum (Fig. 7). Vertex (Fig. 7) irregularly quadrangular, shorter in middle than the maximum width in dorsal view, disc of vertex depressed, with median carina; anterior margin obtusely convex, posterior margin obtusely concave, lateral margins paralleled. Gena (Fig. 8) with one obvious ocellus between compound eye and antenna on each side in lateral view. Frons (Fig. 9) irregularly hexagonal, nearly flat, longer in middle than its maximum width, median carina stout and lateral carinae thin; without verrucae along basal margin and lateral margins; basal margin and frontoclypeal suture arched concaved, lateral margins not paralleled, the base narrow, the maximum width below level of antenna. Clypeus (Fig. 9) triangular, with stout median carina. Rostrum reaching mesotrochanters. Pronotum (Fig. 7) triangular, median carina stout, lateral carinae present, with sunken pits along median carina, anterior margin right-angle concaved, posterior margin straight. Mesonotum (Fig. 7) triangular, median carina obvious, lateral and sub-lateral carinae obscure. Forewings (Fig. 10) oblong, anterior and posterior margin nearly paralleled, apical margin relatively acute, longitudinal veins obvious, without obvious hypocostal plate; ScP long, reaching apical margin, nearly parallel with RP, ScP and RP have a common ScP+RP base, RP not forked, MP forking before middle of forewing, CuA forked into two branches near middle of forewing, CuP present, Pcu and A_1_ uniting near middle of clavus, clavus almost 4/5 of forewing. Hindwings (Fig. 11) well developed, three-lobed, Sc+RP have a common stem, forked near apical part, MP simple, not forked, CuA forked into branches CuA_1_ and CuA_2_ near apical part, CuA_2_ and CuP fused apically, with one transverse vein between RP and MP, MP and CuA_1_, Pcu and A_11_ anastomosing at a medium distance, Pcu, A_11_ and A_12_ simple, non-branched, A_2_ lobe developed, with A_2_ vein simple. Hind tibiae each with two lateral spines near distal half.

***Male genitalia*.** Anal tube (Fig. 13) elongate, longer than the maximum width in dorsal view. Anal style (Fig. 13) short, located near base, not surpassing the end of anal tube. Pygofer (Fig. 12) symmetrical, irregularly rectangular and broad, anterior and posterior margins parallel. Genital styles (Fig. 12) symmetrical, irregularly triangular in lateral view, anterodorsal and ventral margin nearly parallel, the width ca. 2.0 times than its height, bearing process near base of neck, neck of capitulum slender (Fig. 14). Phallobase (Fig. 15) symmetrical, shallowly “U”-shaped and tubular, stout, dorsal lobe developed with hooked process in lateral view. Aedeagus (Fig. 15) symmetrical, with one process in lateral view.

**Figures 17–27.**
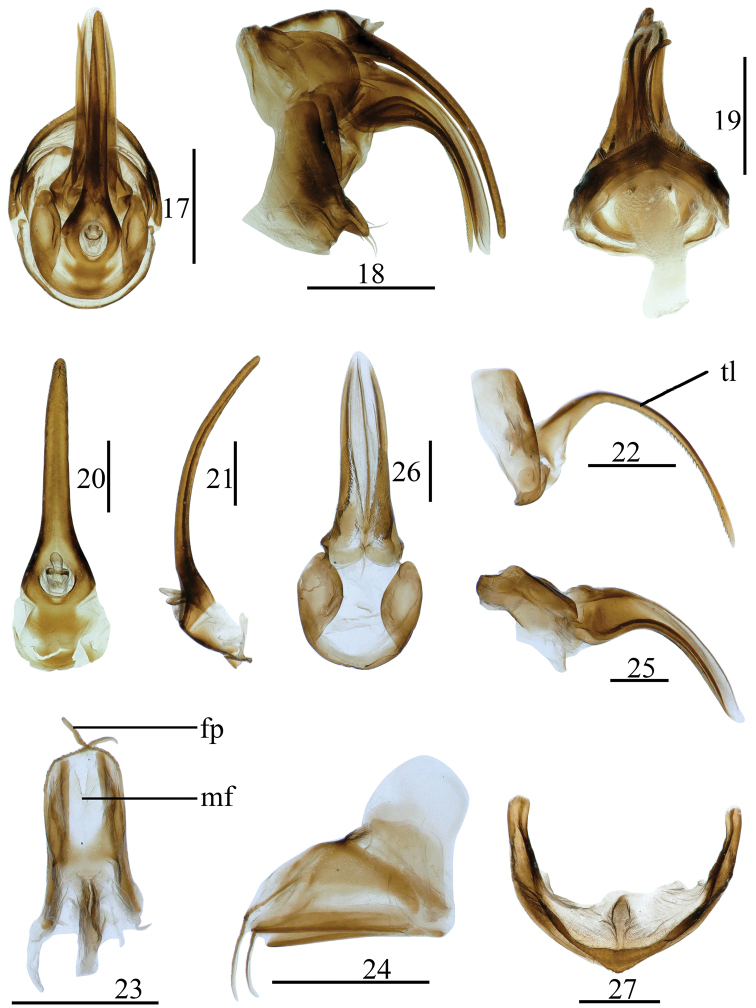
*Tempsarimabipunctata* Chang & Chen, sp. nov. **17** female genitalia, dorsal view **18** same, lateral view **19** same, ventral view **20** female anal segment, dorsal view **21** same, lateral view **22** anterior connective lamina of gonapophyses VIII, lateral view **23** posterior connective lamina of gonapophyses IX, dorsal view **24** same, lateral view **25** gonoplacs, lateral view **26** same, dorsal view **27** sternite VII, ventral view. Scale bars: 1 mm (**17–19**), 0.5 mm (**20–27**). Abbreviations: tl–teeth in inner lateral margin, mf–median field, fp–flagelliform process.

***Female genitalia*** (Figs 17–27). Anal tube (Fig. 20) sclerotized, extremely narrow, and obviously longer in middle line than the width, tapering in dorsal view. Anal style (Figs 17, 20) long or short, located in base of anal tube, not surpassing the end of anal tube. Hind margin of gonocoxa VIII with endogonocoxal lobe not obvious, endogonocoxal process reduced, fused with anterior connective lamina of gonapophyses VIII (Fig. 22). Anterior connective lamina of gonapophyses VIII (Fig. 22) symmetrical, strongly sclerotized, extremely narrow, long, saw-like. Posterior connective lamina of gonapophyses IX (Figs 23, 24) symmetrical, triangular, ventroposterior lobes with long flagelliform process. Gonoplacs (Figs 25, 26) symmetrical, elongate, sclerotized, tuber and tapering in lateral view; the basal part fused in dorsal view. Hind margin of sternite VII convex, with prominence in middle area in ventral view (Fig. 27).

##### Distribution.

China (Hainan).

##### Etymology.

The generic name is derived from a free combination between the genus names *Tempsa* Stål, 1866 (referring to the similar female genitalia) and *Sarima* Melichar, 1903 (type genus in Sarimini). The gender is feminine.

##### Remarks.

The new genus markedly differs from the other genera in Sarimini: 1) frons smooth, with medical carina stout, reaching frontoclypeal suture (Fig. 9); 2) forewing with ScP vein long, reaching apical margin of forewings (Fig. 10); 3) male genitalia with genital styles irregularly triangular in lateral view, the width ca. 2.0 times the height (Fig. 12); 4) apical part of dorsal lobe of phallobase with hooked process (Fig. 15); 5) female genitalia with anal tube extremely narrow and long (Fig. 20), anterior connective lamina of gonapophyses VIII heavily sclerotized, long saw-like (Fig. 22), apical part of posterior connective lamina of gonapophyses IX with flagelliform process (Fig. 23), gonoplacs elongate, tubular in lateral view (Fig. 25).

#### Tempsarima bipunctata

Taxon classificationAnimaliaHemipteraIssidae

Chang & Chen sp. nov.

89DAE230-5EF1-56EB-A48B-656382F6582B

http://zoobank.org/DE35755E-7CFB-49A2-A162-295600A2EFA4

Figures 5–16 , 17–27

##### Type material.

***Holotype***: ♂, China: Hainan, Bawangling (22°28'N, 106°57'E), 13 March 2014, S-Y Xu and J-K Long leg.; paratypes: 10♂♂ 3♀♀, Hainan, Bawangling (22°28'N, 106°57'E), 30 April 2017, L-J Yang leg.; 4♀♀, Hainan, Diaoluoshan (18°39'N, 109°55'E), 15 April 2017, L-J Yang leg.

##### Diagnosis.

This new species is distinguished by the following characters: vertex with four black brown bands along lateral margins and median carina (Fig. 7); mesonotum with each other one dark spot between lateral and sublateral carinae (Fig. 7); genital styles with irregular triangular prominence near dorsal margin at base of capitulum (Fig. 12a); dorsal lobe of phallobase with one small claviform process in base (Fig. 15b), and convex protrusion near middle (Fig. 15c) and apical part with a duckbill-like process (Fig. 15d), lateral margin with one long hooked process (Fig. 15e) on each side; ventral lobe with apical part mushroom-like in ventral view (Fig. 16h); aedeagus with one short hooked process near apical 1/3 in lateral view, directing to cephalad (Fig. 15i).

##### Description.

Body length (from apex of vertex to tip of forewings): male 7.80–8.31 mm (*N* = 11), female 8.80–9.30 mm (*N* = 7); forewing: male 6.50–7.80 mm (*N* = 11), female 7.60–9.30 mm (*N* = 7).

***Coloration*.** General colour yellow-green (Fig. 5). Vertex (Fig. 7) yellow-brown, with four black brown bands along lateral margins and median carina, with pale yellow median carina. Frons and clypeus (Fig. 9) ochreous. Compound eyes black brown, ocelli pale ochreous (Fig. 8). Pronotum and mesonotum (Fig. 7) yellow brown, mesonotum with pair of dark spots between lateral carinae and sublateral carinae. Male forewings (Fig. 6) yellow green, with diffusely brownish irregular speckles near middle of MP vein and CuA vein, and the base of ScP+RP vein; female forewings brown. Hindwings transparent. Abdomen pale yellow-green, suffused with black-brown near middle line. Male genitalia pale yellow green. Female genitalia brown black. Tip of spines on hind tibiae and tarsi black.

***Head and thorax*.** Head (Fig. 7) including eyes distinctly narrower than pronotum (0.77: 1.00). Vertex (Fig. 7) slightly shorter in middle than the width (0.75: 1.00). Frons (Fig. 9) longer in middle than the maximum width (1.14: 1.00), with median carina nearly reaching frontoclypeal suture, sublateral carinae obscure, nearly reaching the level of middle of frons. Pronotum (Fig. 7) shorter in midline than the width (0.31: 1.00). Mesonotum (Fig. 7) shorter in midline than its width (0.43: 1.00); the basal part forked, scutellum sunken. Forewings (Fig. 10) longer than width (2.33: 1.00), MP two branches near basal 1/3, MP_1_ dividing two branches in distal 1/4, MP_2_ not forked, CuA forked into two branches in middle of forewing, Pcu and A_1_ uniting slightly after MP bifurcation. Hindwings (Fig. 11) with one transverse vein between CuP and Pcu near apical part, with transverse vein between Pcu+A_11_ and A_12_ near basal 1/3. Spinal formula of hind leg (2)7/6, 7/2.

***Male genitalia*.** Anal tube (Fig. 13) longer than its widest breath (2.90: 1.00) in dorsal view, anterior margin arched convex, lateral margins nearly parallel at apical 2/3, the basal 1/3 part broader than apical part. Anal style (Fig. 13) small, extremely short and thin, located in basal 2/5 of anal tube, not surpassing the end of the anal pore. Pygofer (Fig. 12) with dorsal and ventral margin paralleled in lateral view. Genital styles (Fig. 12) with irregular triangular prominence at base of capitulum (Fig. 12a). Capitulum of genital styles irregularly keen-edged triangular, neck very long and obvious (Fig. 14). Phallobase (Figs 15, 16) with dorsal margin of dorsal lobe with one small claviform process in base (Fig. 15b) in lateral view, convex protrusion near middle (Fig. 15c) and apical part with duckbill-like process (Fig. 15d), lateral margin with one long hooked process on each side (Figs 15e, 16e), surpassing middle of phallobase, directing to cephalad, and lateral margin waved obviously, with one lobe-like process (Fig. 15f); lateral lobe splitting into two branches, slightly shorter than the dorsal lobe, with unobvious small lamina-like process (Fig. 15g); ventral lobe slightly shorter than lateral lobe in lateral view, stout, with apical part mushroom-like (Fig. 16h) in ventral view. Aedeagus (Figs 15, 16) with one short hooked process on each side (Figs 15i, 16i) near apical 1/3 in lateral view, directing to cephalad.

***Female genitalia*.** Anal tube (Figs 17, 20) longer in middle line than the width (3.15: 1.00), the basal 1/3 part broader, inclined to ventral margin in lateral view (Fig. 21). Anal style (Figs 20, 21) long and stout, located in basal 1/6 of anal tube, surpassing the end of anal pore. Anterior connective lamina of gonapophyses VIII (Figs 18, 22) extremely long saw-like, with a row of teeth in inner lateral margin (Fig. 22: tl). Posterior connective lamina of gonapophyses IX (Figs 23, 24) relatively broad in dorsal view, with lateral field and sublateral field unobvious, membranous, median field membranous, with deep sunken (Fig. 23: mf), apical part of ventroposterior lobes with long flagelliform process (Fig. 23: fp). Gonoplacs (Fig. 25) irregularly triangular, tapering, apical part membranous, thin tuber in latera view; fused dorsally to form a sheath surrounding the anterior connective lamina of gonapophyses VIII (Fig. 26). Hind margin of sternite VII with distinctly triangular prominence in middle area in ventral view (Fig. 19), inner margin with membranous process (Fig. 27).

##### Distribution.

China (Hainan).

##### Etymology.

The species name is derived from a combination of the prefix “bi-” and Latin noun “punctata”, suggesting the paired dark spots of mesonotum.

##### Host plant.

Unknown.

#### Tetrichina

Taxon classificationAnimaliaHemipteraIssidae

Chang & Chen gen. nov.

450245B3-BD25-5772-9275-052C3DCA95DC

http://zoobank.org/24124E55-71B3-4696-9CB9-F5D87D53BC8B

Figures 28–39 , 40–48

##### Type species.

*Tetrichinatrihamulata* Chang & Chen, sp. nov., here designated.

**Diagnosis.** Related to the genus *Sarimodes* Matsumura, 1916, but it is distinguished as follows: frons (Fig. 32) without obvious verrucae along basal and lateral margins (frons with obvious verrucae in *Sarimodes* (Meng and Wang 2016: fig. 18)); forewings (Fig. 33) with ScP long, reaching apical margin of forewing, with short vein in base of ScP (ScP only surpassing middle of forewings, without short vein in *Sarimodes* (op. cit.: fig. 19)); genital styles (Fig. 35) irregularly elliptical in lateral view, neck of capitulum extremely long (genital styles irregularly rounded, neck of capitulum short in *Sarimodes* (op. cit.: fig. 22)).

##### Description.

Body medium size, slightly flat in dorsal view.

**Figures 28–39.**
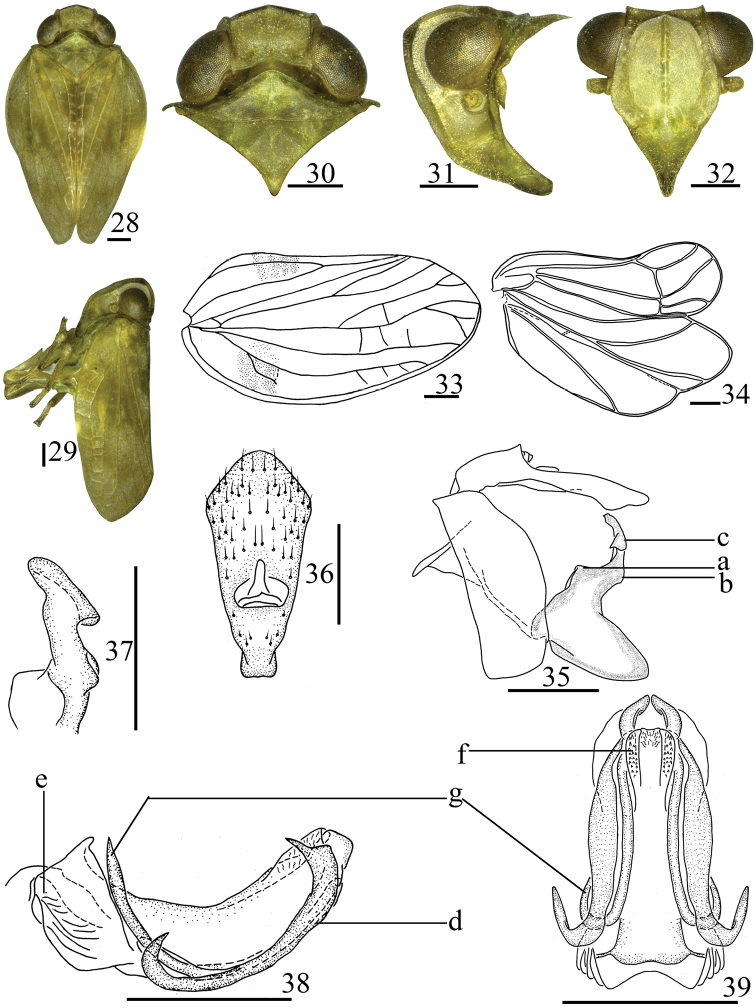
*Tetrichinatrihamulata* Chang & Chen, sp. nov. **28** adult, dorsal view **29** same, lateral view **30** head and thorax, dorsal view **31** same, lateral view **32** head, ventral view **33** forewing **34** hindwing **35** male genitalia, lateral view **36** male anal segment, dorsal view **37** capitulum of genital styles, posterior view **38** aedeagus and phallobase, lateral view **39** same, ventral view. Scale bars: 0.5 mm. Abbreviations: a–obvious triangular prominence, b–unobvious triangular prominence, c–lobed process, d–bidirectional hooked process, e–irregularly quadrangular prominence, f–lobe-like process, g–long hooked process.

***Head and thorax*.** Width of head including eyes narrower than pronotum (Fig. 28). Vertex (Fig. 30) quadrangular, shorter in middle than its maximum width in dorsal view, disc of vertex depressed, median carina obscure, with one pit between median and lateral carinae; anterior margin obtusely convex, posterior margin arched concave, lateral margins paralleled. Gena (Fig. 31) with one obvious ocellus between compound eye and antenna on each side in lateral view. Frons (Fig. 32) irregularly hexagonal, length in midline nearly equal to its maximum breadth; with median and lateral carinae, reaching frontoclypeal suture; without obvious verrucae along basal and lateral margins; basal margin obtusely concaved; frontoclypeal suture slightly arched concave, lateral margins not paralleled; the base narrow, the maximum width below level of antenna. Clypeus (Fig. 32) triangular, with median carina stout, short or long. Rostrum just reaching mesotrochanters. Pronotum (Fig. 30) triangular, with median and lateral carinae, and with two pits between median and lateral carinae, anterior margin obtusely-angle concaved, posterior margin straight. Mesonotum (Fig. 30) triangular, with median and lateral carinae, sublateral carinae obscure. Forewings (Fig. 33) irregularly oval, anterior margin distinctly arched convexly, posterior margin straight, apical margin distinctly arched, longitudinal veins obvious, with a few unobvious short transverse veins, without hypocostal plate; ScP long, reaching apical margin, ScP forked one short vein near base, ScP and RP have a common ScP+RP base, RP simple, not forked, MP and CuA forked into two branches near middle of forewing, CuP present, Pcu and A_1_ uniting near base 2/3 of clavus, clavus almost 4/5 of forewing. Hindwings (Fig. 34) well-developed of typical Sarimini type, three lobes, ScP+PR have a common stem, forked near apical part, MP simple, not forked, CuA forked into branched CuA_1_ and CuA_2_ near apical part, CuA_2_ and CuP fused apically, with one transverse vein between RP and MP, MP and CuA_1_, Pcu and A_11_ anastomosing at medium distance, Pcu, A_11_, and A_12_ not branched, A_2_ lobe relatively narrow, A_2_ vein simple. Hind tibiae each with two lateral spines near distal half.

***Male genitalia*.** Anal tube (Fig. 36) irregularly pentagonal, longer in middle than its widest breadth in dorsal view, basal part extremely narrow, apical part broad, the maximum width near the apical part. Anal style (Fig. 36) relatively long, not surpassing the end of anal tube. Pygofer (Fig. 35) symmetrical, irregularly rectangular in lateral view, dorsal and ventral margin paralleled. Genital styles (Fig. 35) irregularly elliptical in lateral view, postero-dorsal margin long and nearly parallel to ventral margin, bearing process near base of neck. Capitulum (Fig. 37) extremely developed, neck of capitulum extremely long. Phallobase (Figs 38, 39) symmetrical, U-like tube in lateral view, apical part of dorsal lobe with hooked processes on each side in lateral view. Aedeagus (Figs 38, 39) with one hooked process on each side in lateral view.

**Figures 40–48.**
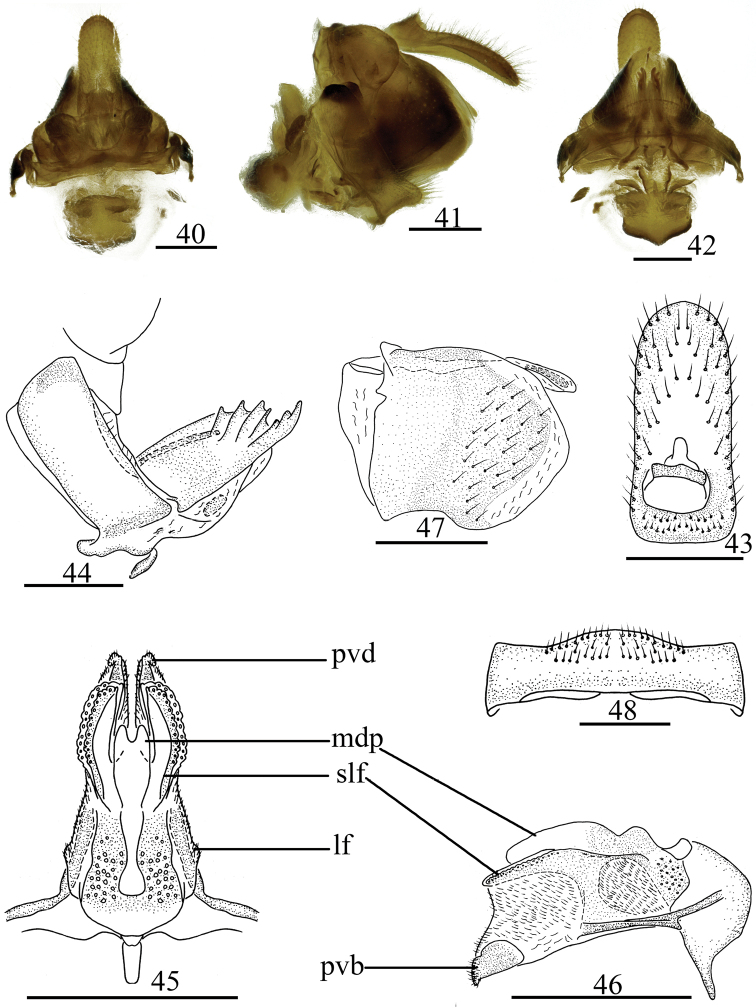
*Tetrichinatrihamulata* Chang & Chen, sp. nov. **40** female genitalia, dorsal view; **41** same, lateral view **42** same, ventral view **43** female anal segment, dorsal view **44** anterior connective lamina of gonapophyses VIII, lateral view **45** posterior connective lamina of gonapophyses IX, dorsal view **46** same, lateral view **47** gonoplacs, lateral view **48** sternite VII, ventral view. Scale bars: 0.5 mm. Abbreviations: lf–lateral field of posterior connective lamina of gonapophyses IX; slf–sublateral field of posterior connective lamina of gonapophyses IX; mdp–medial dorsal process; pvd–posterior ventral lobes.

***Female genitalia*** (Figs 40–48). Anal tube (Figs 40, 43) elongate, longer in middle line than its width. Anal style (Fig. 43) long, located near base of anal tube, not surpassing the end of anal tube. Anterior connective lamina of gonapophyses VIII (Fig. 44) irregularly rectangular, with four keeled teeth in lateral group and three large teeth in apical group. Posterior connective lamina of gonapophyses IX (Figs 45, 46) triangular and narrow in dorsal view. Gonoplacs (Fig. 47) irregularly round, without keels. Hind margin of sternite VII with prominence in middle area in ventral view (Fig. 48).

##### Distribution.

China (Hainan).

##### Etymology.

The generic name is derived from the arbitrary combination of generic name “*Tetrica*” and word “China”. The gender is feminine.

#### Tetrichina trihamulata

Taxon classificationAnimaliaHemipteraIssidae

Chang & Chen sp. nov.

FEFF6315-1DEF-53AF-B474-D9161729B06F

http://zoobank.org/F3785430-C2D3-402E-9A04-85744F64F56A

Figures 28–39 , 40–48

##### Type material.

***Holotype***: ♂, China: Hainan Province, Jianfengling National Park (18°42'N, 108°51'E), 20 April 2014, W-C Yang leg.; paratypes: 3♂♂ 2♀♀, data same as holotype; 5♂♂, Hainan, Jianfengling (18°42'N, 108°51'E), 13–16 January 2011, J-K Long and P Zhang; 2♂♂2♀♀, Hainan, Bawangling National Nature Reserve (22°28'N, 106°57'E), 7–11 January 2011, J-K Long and P Zhang; 2♂♂, Hainan Province, Datian National Nature Reserve (19°06'N, 108°47'E), 12–13 April 2013, J-K Long, J-C Xing and Y-B Zhang leg.

##### Diagnosis.

This new species looks like *Sarimodesclavatus* Meng & Wang, 2016 (Meng and Wang 2016: figs 17–32), but differs from the latter by: 1) vertex shorter in middle line than its maximum width, but longer in *S.clavatus*; 2) capitulum of genital styles with anterior margin with one triangular prominence near base, but in *S.clavatus* without triangular prominence; 3) phallobase with dorsal lobe with one stout bidirectional hooked process in lateral view; but with one hooked process in *S.clavatus*.

##### Description.

Body length: male 5.02–5.64 mm (*N* = 13), female 5.73–5.82 mm (*N* = 4); forewing: male 4.13–4.57 mm (*N* = 13), female 4.70–4.88 mm (*N* = 4).

***Coloration*.** General colour yellow-green (Figs 28, 29). Compound eyes brown, ocelli pale green (Fig. 31). Forewings (Fig. 28) yellow-green, with diffuse brownish irregular speckles near middle. Tip of spines on hind tibiae and tarsi black.

***Head and thorax*.** Head (Fig. 30) including eyes narrower than pronotum (0.76: 1.00). Vertex (Fig. 30) shorter in middle than the width (0.63: 1.00), median carina liner. Frons (Fig. 32) slightly longer in middle than its maximum breadth (1.02: 1.00), median carina stout, lateral carinae slender. Pronotum (Fig. 30) shorter in midline than the width (0.24: 1.00). Mesonotum (Fig. 30) shorter in midline than the width (0.30: 1.00). Forewings (Fig. 33) longer than width (2.00: 1.00), RP simple, reaching apical margin, MP two branched near middle, MP_1_ and MP_2_ forked near distal part, CuA forked into two branches in middle of forewing, paralleling MP bifurcation, Pcu and A_1_ uniting slightly before MP bifurcation. Hindwings (Fig. 34) without transverse vein between Pcu+A_11_ and A_12_. Spinal formula of hind leg (2)8/6, 10/2.

***Male genitalia*.** Anal tube (Fig. 36) longer in middle than its widest breath (2.40: 1.00) in dorsal view, anterior margin obtuse convex, the base extremely narrow, the width near apical 1/4. Anal style (Fig. 36) thin, located near middle, surpassing the end of anal pore. Pygofer (Fig. 35) with anterior margin straight, posterior margin arched convex in lateral view. Genital styles (Fig. 35) with antero-dorsal margin short, anterior margin bearing obvious triangular prominence (Fig. 35a) and posterior margin bearing unobvious triangular prominence (Fig. 35b) near base of capitulum. Capitulum with of genital styles irregular triangular, with irregular lobed process in basal of capitulum (Fig. 35c), neck of capitulum extremely stout (Fig. 37). Phallobase (Figs 38, 39) with dorsal lobe simple, apical part membranous, in lateral view, with one stout bidirectional hooked process (Fig. 35d) on each side, one short directing to anterior-dorsad, one relatively long, directing to posterior-dorsad; ventrolateral lobe with irregularly quadrangular prominence (Fig. 35e) in basal 1/3 in lateral view; lateral lobe splitting into two branches, more longer than dorsal lobes; ventral lobe shorter than lateral lobe in lateral view, apical part with lobe-liked process (Fig. 39f) in ventral view. Aedeagus (Figs 38, 39) with one extremely long hooked process on each side (Fig. 38g) in lateral view, directing to cephalad (Fig. 39g).

***Female genitalia*.** Anal tube (Figs 40, 43) longer in middle line than the width (2.10: 1.00), apical margin arched convex, lateral margins paralleled. Anal style (Fig. 43) relatively long and stout, located in basal 1/4 of anal tube, surpassing the end of anal pore. Gonocoxa VIII relatively long and narrow, gonocoxa VIII with endogonocoxal lobe obvious, with one small claviform sclerotic process, endogonocoxal process membranous and developed (Fig. 44). Anterior connective lamina of gonapophyses VIII (Fig. 44) with four keels leading to four teeth in lateral group and three teeth in series in apical group. Posterior connective lamina of gonapophyses IX (Figs 45, 46) narrow, sub-triangular in dorsal view, lateral field membranous developed, with triangular membranous process with microvilli (Fig. 45: lf); sub-lateral field developed and sclerous, with the inner margin waved (Fig. 45: slf); median field with symmetric goblet-shaped process, apical margin in middle concave (median dorsal process) (Fig. 45: mdp); distal parts bent at obtuse angled in dorsal view (posterior ventral lobes) (Fig. 45: pvd). Hind margin of sternite VII obviously convex in medial area in ventral view (Figs 42, 48).

##### Distribution.

China (Hainan).

##### Etymology.

The species name is derived from a combination of the prefix “tri-” and Latin noun “hamulata”, referring to the phallobase and aedeagus with three variously hooked processes.

##### Host plant.

Unknown.

## Discussion

Emeljanov (1990) proposed two types of female genitalia along with different functions: the piercing-type in order to pierce plant tissue for laying eggs, and the raking-type in order to cover eggs with secretions of female genitalia. Bourgoin (1993) also characterized two types of female genitalia in Fulgoroidea along with their morphology: the plesiomorphic orthopteroid-type, such as species of Cixiidae, Delphacidae, and Kinnaridae, and the derived fulgoroid-type, such as in *Metaphaenabasilactea* (Dictyopharidae) and other planthopper families including Issidae. In the family Issidae, most of the groups have one common type of female genitalia, which is of the representative raking-type based on fulgoroid-type structural morphology: anterior connective lamina of gonapophyses VIII irregularly rectangular, rake-like, with developed endogonocoxal process, gonoplacs rounded and membranous, as in *Tetrichinatrihamulata* Chang & Chen, sp. nov. (Figs 44–47). However, in several other Issidae, another kind of female genitalia is observable with anterior connective lamina of gonapophyses VIII strongly sclerotized and narrow, bearing a row of teeth, endogonocoxal process short and degraded, the apical part of posterior connective lamina of gonapophyses IX flagelliform, gonoplacs elongate, beak-shaped and sclerotized. This type of female genitalia belongs to the fulgoroid-type from which it is derived but with a shift of the raking function, probably returning to a secondary piercing one. This type is already recorded in IssidaeHysteropterinae in *Euplilis* Walker, 1857, *Gabaloeca* Walker, 1870, and also in the Sarimini genus *Tempsa* Stål, 1866 (Gnezdilov 2013), and *Tempsarima* Chang & Chen, gen. nov. also belongs to this type. It is also known in Nogodinidae, but gonoplacs are round in *Ugoa* Fennah, 1945 and *Jamaha* Gnezdilov & O’Brien, 2008, while beak-shaped in *Caudibeccuscarlota* (Myers) (Gnezdilov 2013). The same tendency is also observed in anterior connective laminae in the genera *Colpoptera* and *Caudibeccus* (Gnezdilov 2013: figs 17, 22). Gnezdilov (2013) proposed the term “styletization” standing for the tendency of narrowing and referring to the secondary piercing-fulgoroid type of female genitalia. The irregular triangular gonoplacs of *Colpopterasinuata* Burmeister might represent a distinct transition from the rounded to the elongate beak-shaped type. In the tribe Sarimini, a similar transition is observable with *Microsarimodes* Chang & Chen, 2019 bearing irregular triangular gonoplacs (Chang et al. 2019: fig. 36), and the distal parts of the posterior connective laminae of gonapophyses IX slender and narrowing (Chang et al. 2019: fig. 34), standing for the transition from a non flagelliform to flagelliform conformation.

## Supplementary Material

XML Treatment for Tempsarima

XML Treatment for Tempsarima bipunctata

XML Treatment for Tetrichina

XML Treatment for Tetrichina trihamulata
